# Significance of the Intraoperative Methylene Blue Test for Postoperative Evaluation of the Vesicourethral Anastomosis

**DOI:** 10.1155/2012/702412

**Published:** 2012-08-12

**Authors:** J. N. Nyarangi-Dix, S. Pahernik, J. L. Bermejo, L. Prado, M. Hohenfellner

**Affiliations:** ^1^Department of Urology, University Hospital of Heidelberg, 69120 Heidelberg, Germany; ^2^Department of Medical Biometry and Informatics, Heidelberg University, Heidelberg, Germany

## Abstract

We prospectively investigated whether routine evaluation of the vesicourethral anastomosis (VUA) after radical prostatectomy can be waived. Primary integrity of the VUA was analysed by an intraoperative methylene-blue test (IMBT) and postoperatively by conventional cystography. Data on the IMBT, contrast extravasation and prostate volume as well as pad usage were collected prospectively. Significantly more patients with a primary watertight anastomosis demonstrated by the MBT had no leakage in the postoperative cystography (*P* < 0.001). In a multivariate logistic regression with adjustment for prostate size and surgeon, the positive correlation between IMBT and postoperative cystography remained statistically significant (*P* = 0.001). The IMBT is easy to perform, inexpensive, and timesaving. With it postoperative evaluation of VUA for integrity can be waived in a significant number of patients. Following our algorithm, the Foley can be removed without further testing of the VUA, whenever the IMBT detected no leakage.

## 1. Introduction

Radical prostatectomy (RP) is one of the major therapeutic options in the management of organ confined prostate cancer [1]. In order to support healing of the vesicourethral anastomosis (VUA) after RP, temporary urinary drainage with a transurethral Foley catheter is routinely performed [1]. This catheterisation is usually continued for the first 4–14 days after the procedure [1–4]. Before removal of the Foley, it is recommended to rule out anastomotic leakage [2]. This makes it often necessary for the patient to revisit his attending physician, for example, for performance of a conventional cystography [2, 3]. This is not only time consuming for all parties involved but also associated with higher costs and patient discomfort [5]. For this reason, we initiated a prospective study aimed at investigating whether cystography studies before Foley removal can be somehow reduced or in certain cases entirely waived. The study was performed according to ethical principles for medical research as noted in the current Declaration of Helsinki and was in accordance with the German Medical Association's professional code of conduct. All patients gave informed consent for participation in this study.

## 2. Material and Methods

We prospectively investigated 103 consecutive men undergoing radical retropubic prostatectomy in our institution. All patients were followed up at 0, 3, 6, and 12 months. RP was performed in our institution by 4 skilled surgeons each performing the procedure as described by Walsh et al. [1]. We analysed the primary water tightness of the VUA by filling up the bladder after tying the anastomosis. In order to ease detection of even minimal anastomotic leakage, a sterile solution made of 5 mL methylene blue and 95 mL normal saline was prepared. White sterile dressings were placed around the VUA to further ease identification of methylene blue leakage. A syringe was used to manually instil 100 mL of the prepared methylene blue solution into the bladder via the routinely placed 20 French transurethral Foley. The methylene blue solution remained instilled in the bladder for 15 s, before passive drainage by opening of the Foley was allowed. A primary watertight anastomosis was defined as lack of leakage of the methylene blue solution verified by the unstained white dressings. The methylene blue solution cost us only 1.19 Euros. 

In order to assure integrity of the VUA, we performed a conventional cystography test in all patients on postoperative day (POD)-5. Cystographies were independently analysed by an urologist and a radiologist. These analysts had no knowledge of the result of the intraoperative methylene blue test (MB test). Only studies clearly identified by both analysts were classified as such. It was necessary for both analysts to independently identify lack of contrast extravasation for a cystography to be categorised as such. A patient was classified to the extravasation group, if at least one analyst identified contrast leakage in the respective cystography. As routinely practised in our institution at the time of the study, the minimum time of catheterisation after prostatectomy was 12 days. Whenever necessary, the patients were readmitted in the clinic for a repeat cystography test on POD 12–16. Catheterisation was continued when any form of anastomotic leakage was identified and the cystography test repeated weekly thereafter until integrity of the anastomosis was verified by both analysts. 

We collected data on characteristics like age at the time of operation, tumour stage, Gleason score (GS), surgical margin, preoperative PSA (prostate specific antigen) value, prostate volume as measured in a preoperative transrectal ultrasound, anastomotic strictures, contrast extravasation in the cystography on POD-5, and duration of catheterisation. For evaluation of social continence pad usage at 3, 6, and 12 months after removal of the catheter was documented as well. We classified continence according to the definition of the European Association of Urology (2009) for postprostatectomy continence, that is, patients with usage of none or 1 safety pad per day at the time of evaluation (3, 6, and 12 months after prostatectomy) were classified as being continent [6]. 

The association between the above-mentioned patient characteristics, primary water tightness, and time to catheter removal (within or after two weeks) was explored by logistic regression. Statistical analyses were conducted using SAS. The study had a power of 90% to identify a relative rate higher than 1.1 for the association between primary watertight anastomosis and the result of the postoperative cystography (type I error probability 5%). Descriptive statistics was given with median and range.

## 3. Results

The median age of the patients was 66.3 years (range: 45.3–79.2 years). In the investigated collective of 103 men, 71 (68.9%) detected no leakage in the IMBT. Among these patients with a primary watertight VUA, 83.1% (*n* = 59) had no contrast extravasation in the POD-5-cystography. On the other hand, only 37.5% (*n* = 12) of patients with leakage in the IMBT detected no contrast extravasation at the same time (Table 1). We found a significantly higher proportion of patients with no leakage in the POD-5-cystography had an intraoperatively watertight anastomosis compared to those with intraoperative methylene blue leakage (83.1 versus 16.9%, *P* = 0.001). The sensitivity and specificity of the IMBT were 83.1% and 62.5%, respectively (Table 2). 68.9% of all cystography studies demonstrated a watertight VUA. Taking the postoperative POD-5-cystography as a validated technique for evaluating integrity of the VUA, the IMBT reached positive and negative predictive values of 83.1 and 62.5%, respectively. 16.9% of IMBTs were falsely positive, that is, demonstrated leakage when there was no relevant extravasation on POD-5 and 32.5% were falsely negative, that is, showed no intraoperative leakage while there was minimal extravasation on the POD-5-cystography (Table 2). There was significant correlation between results of the IMBT and POD-5-cystography (*P* < 0.001). In this study, a majority of the patients were accurately classified by the IMBT, that is, 76.7% on POD-5. Interestingly, all men with an intraoperatively watertight VUA ascertained by the IMBT demonstrated no more contrast extravasation at POD-12. 

**Figure 1:**
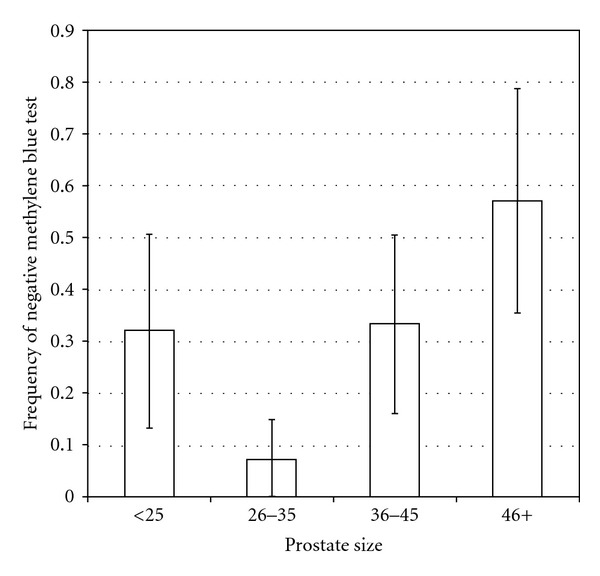
Relationship between prostate size and negative IMBT (i.e., no leakage).

Intraoperative water tightness negatively correlated with the prostate size (*P* = 0.01). While the anastomosis was intraoperatively watertight in 68% of the men with prostates smaller than 25 mL, this was the case in only 43% of those with prostates larger than 45 mL (Figure 1). We found no significant difference in the rate of IMBT regarding patient age, tumour stage, GS, surgical margins, PSA value, and neurovascular bundle preservation. On the other hand, a borderline statistically significant difference in the rate of intraoperative water tightness among surgeons was identified (*P* = 0.05). The earlier-mentioned association between IMBT and postoperative cystography remained statistically significant even after adjustment for surgeon and prostate size in a multivariate logistic regression (*P* = 0.001).

12 months after RP, 91.9% of the patients were continent according to the EAU definition, that is, usage of none or a safety pad per day. The intraoperative water tightness according to the IMBT and continence rates at 3 (*P* = 0.19), 6 (*P* = 0.17), and 12 months (*P* = 0.11) showed no significant association (Table 1).

**Figure 2:**
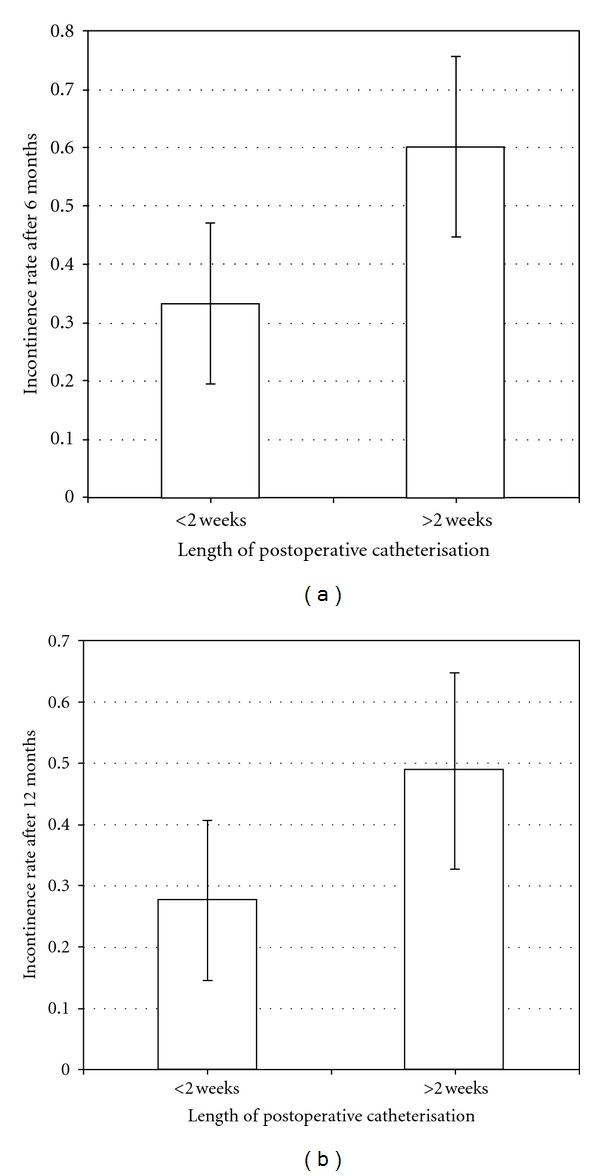
Relationship between time of catheter removal and incontinence rates 6 and 12 months after operation.

The median time of postoperative catheterisation was 14 (range 12–30) days. In 59.2% of the patients, the Foley was removed within the first 2 weeks of the procedure. We found that postoperative catheterisation was significantly shorter in smaller prostates (*P* = 0.02). The Foley was removed within two weeks in 52% of men with a prostate volume <25 mL compared to 33% in men with a prostate size >45 mL. Catheterisation was also significantly shorter in case of neurovascular bundle preservation (73.5% versus 52.2%, *P* = 0.04). As far as continence rates are concerned, they lied by 89.6% and 94% at 6 and 12 months, respectively, and were significantly higher when the Foley was removed within the first 2 weeks after prostatectomy (*P* = 0.01 and 0.05, resp.). These rates were on the other hand significantly lower, that is, 75% and 84.4% if the period of catheterisation was longer than 2 weeks (Figure 2). We found no statistical differences in the length of postoperative catheterisation as far as continence rates at 3 months, patient age, tumour stage, GS, surgical margin, PSA value and the surgeon were concerned. 

2 cases (1.9%) of anastomotic strictures occurred during the study period. 1 patient had a primary watertight anastomosis in the IMBT and the other did not. Both patients underwent routine internal urethrotomy in our clinic and were both continent 12 months after the prostatectomy. No major complication was observed during the study period.

## 4. Discussion

In order to increase patient comfort and facilitate recuperation, the duration of Foley catheterisation after RP has successively decreased over the last decades [2–4, 7, 8]. The length of postoperative catheterisation often correlates with the duration of urinary extravasation that on the other hand seems to inversely correlate with anastomotic strictures and continence rates [9, 10]. Even though some authors propose removal of the Foley without prior evaluation of urinary extravasation, this practice has not yet found wide acceptance among urologists [11]. Before Foley removal most institutions routinely investigate anastomotic extravasation, for example, with a cystography, making it often necessary, for the recuperating patient to revisit his attending physician [2–4, 8]. The organisation around and performance of a cystography are not only time consuming for all parties involved but also associated with higher costs and patient discomfort [5, 11]. Depending on institutional practice, the Foley is usually removed when there is minimal anastomotic leakage or none at all [2, 4, 5, 8]. Our institution prefers the latter practice. A number of studies have investigated parameters that predict postoperative anastomotic leakage. So far no validated predictive parameters of postoperative urinary extravasation have been identified that make postoperative proof of anastomotic integrity before Foley removal unnecessary [12]. For this reason, we initiated this prospective study to investigate whether cystography studies for evaluation of the VUA before Foley removal can be minimised or even entirely waived. 

**Figure 3:**
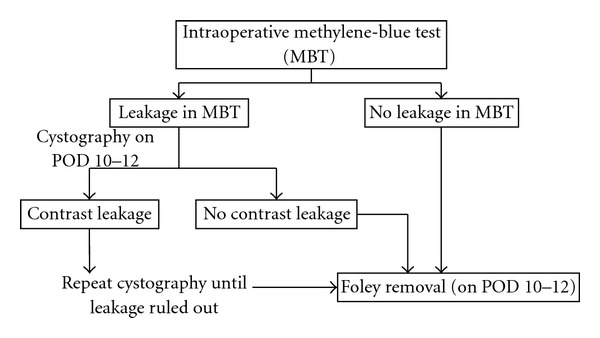
Algorithm for routine workup after intraoperative methylene blue test (IMBT).

69% of all our patients had a watertight anastomosis identified by the IMBT. The majority (83%) of these men had a watertight VUA with no contrast extravasation in the POD-5-cystography. Only a small fraction (17%) of these patients had minimal leakage in the POD-5-cystography. Statistically, the rate of VUA integrity demonstrated in the POD-5-cystography was significantly higher in cases in which the IMBT detected a watertight VUA (*P* < 0.001). At the same time, contrast extravasation in the cystography was significantly higher in those cases where the IMBT detected leakage (*P* < 0.001). The sensitivity and specificity of the IMBT were with 83.1 and 62.5%, respectively, high. If we had applied less strict rules and/or had a single analyst interpreting cystography studies as most institutions do, these rates may have been much higher. In addition to that, the cystography study that we adopted as our validating study was performed on POD-5, which is earlier than most authors [1, 4, 5, 8, 12]. All cases, in which the IMBT was watertight, demonstrated no contrast leakage by POD-12 at the latest. Based on these results, the Foley could have been safely removed without additional cystography in all patients with a watertight VUA verified by the intraoperative methylene blue test. In this case we could have waived a cystography study in over two-thirds of our patients. According to our study design, no cystography was performed between POD-5 and 12. It is therefore not possible to pinpoint the exact day when no more urinary leakage was present in all cases. This would have been especially interesting in those men with persistent extravasation on the POD-5-cystography. We therefore suggest application of the algorithm shown on Figure 3 that can be routinely applied irrespective of institution-preferred period of catheterisation. It can most certainly be presumed that an even shorter catheterisation is possible as currently routinely practiced in most institutions including our own. Further studies addressing this question are pending.

Intraoperative water tightness as analysed by the IMBT also significantly correlated with the size of the prostate (*P* = 0.01). In the latter case, we found a significantly higher rate of primary watertight VUA in smaller prostates (Volume < 25 mL). We suppose that the reason for this could be that the removal of a small prostate leaves a shorter distance to bridge while doing the anastomosis and therefore possibly less tension on the VUA allowing for more watertight anastomoses. This hypothesis cannot be validated though since it was not primary subject of our study; further evaluation is still pending. We also identified a borderline surgeon-specific difference in the rate of intraoperative water tightness (*P* = 0.05). Since all involved surgeons had a similar level of experience, we could not identify the reason for this discrepancy that barely reached statistical significance. To our knowledge, no study so far has found a correlation of prostate size to the integrity of the VUA or investigated the influence of the surgeon on the VUA. The relevance of this knowledge is at this point unknown. 

As far as patient age, tumour stage, GS, surgical margin, PSA value, and neurovascular bundle preservation are concerned, we found no significant difference in the rate of intraoperative water tightness.

Notwithstanding the strict rules applied in the interpretation of our postoperative cystography studies, our overall rate of contrast extravasation on POD-5 was comparable to that in other series that identified postoperative leakage in 21–31% of the cases [4, 9, 10]. In this analysis, the mean and median time of postoperative catheterisation was longer than that reported in other series. This was due to our clinical practice at the time initiation of the study and formulation of the study protocol with a minimum period of catheterisation of 12 days. An additional factor was that all cystography studies were strictly interpreted by two independent analysts who had to note an unanimous finding. If this was not the case, catheterisation was continued until leakage was certainly ruled out by both analysts. This practice is contrary to studies from institutions that routinely remove the Foley even when minimal contrast extravasation is present [2, 8, 9, 11–13]. In case of application of the latter practice, an earlier removal of the Foleys would have been possible in the majority of patients in our study collective. 

Interestingly, we found that postoperative catheterisation was significantly shorter in smaller prostates <25 mL (*P* = 0.04) and in case of neurovascular bundle preservation. To our knowledge, this association has not been reported so far and therefore its significance remains unknown. We on the other hand found no significant correlation between the length of catheterisation and patient age, tumour stage, GS, surgical margin, PSA value, or the surgeon. 

12 months after the RP, approx. 92% of all patients were continent. This rate lies in the upper range when compared to those reported in other large series [8, 14, 15]. However, no significant correlation of postoperative continence to the IMBT was identified. Interestingly, continence rates at 6 and 12 months were significantly higher when the Foley was removed within 2 weeks of the procedure and speak for the current trend to shorter catheterisation. These continence rates at 6 and 12 months were approx. 90 versus 75% (*P* = 0.01) and 94 versus 84% (*P* = 0.05), respectively, when catheterisation was maintained for more or less than 2 weeks. These results vary with other studies that report no significant difference in the continence rates even in cases of prolonged catheterisation [8, 16]. Even though this was not the focus of this study, it would be interesting to primarily analyse the role of the duration of catheterisation on continence in a larger series.

Our anastomotic stricture rate (1.9%) was in the lower range of that reported in other series [8]. This is probably related to our clinical practice in which urinary drainage was maintained until integrity of the vesicourethral anastomosis was undoubtedly proven. 

## 5. Conclusions

In conclusion, we find that the IMBT is a promising screening tool that is easy, inexpensive, and timesaving. We suggest routine application as a screening tool to identify patients in whom postoperative evaluation of the VUA can be waived. According to our results the Foley can be safely removed without prior evaluation of the vesicourethral anastomosis when the IMBT demonstrated a watertight anastomosis. Nevertheless, urinary extravasation should still be routinely ruled out in case of intraoperative methylene blue leakage. 

## Figures and Tables

**Table 1:** Relation between patient characteristics and intraoperative methylene blue test.

	IMBT: no leakage	IMBT: leakage	*P* value
CUG-POD 5			
No extravasation	83.1%	16.9%	<0.001*
Extravasation	37.5%	62.5%	
Prostate volume			
≤25 mL	68%	32%	0.01*
>45 mL	43%	57%	
PSA-value			
≤10	67.9%	32.1%	0.81
>10	72%	28%	
Gleason score			
<7	67.9%	32.1%	0.96
≥7	72%	28%	
Incontinence at 3 months			
no	72.9%	27.1%	0.17
yes	58.8%	41.2%	
Incontinence at 6 months			
no	70.8%	29.2%	0.19
yes	57.5%	17.5%	
Incontinence at 12 months			
no	70.3%	29.7%	0.11
yes	53.1%	46.9%	

**Table:** (a)

	Cystography test no leakage	Cystography test leakage	Totals
Methylene blue test no leakage	59	12	71
Methylene blue test leakage	12	20	32

Totals	71	32	

**Table:** (b)

	Cystography test no leakage	Cystography test leakage
Methylene blue test no leakage	83.1% true positive	16.9% false positive
Methylene blue test leakage	37.5% false negative	62.5% true negative
